# Medical Association Meeting

**Published:** 1898-05

**Authors:** 


### Society Reports.

### MEDICAL ASSOCIATION MEETING.

The forty-ninth annual meeting of the Georgia Medical Association was held at Cumberland Island, April 20, 21 and 22, 1898. In the absence of the president, Dr. J. B. Morgan, of Augusta, the meeting was presided over by the vice-president, Dr. L. G. Hardman.

The address of welcome was delivered by Col. R. E. Park; the response on behalf of the association by Dr. Hardman. Dr. J. A. Butts, of Brunswick, represented the local profession in welcoming the visiting members.

The papers on the program were, for the most part, read by title, but the following were presented by the authors:

Dr. J. G. Hopkins—"A Few Cases From My Note-Book." The first case was that of a fistula in the sacral region which communicated with a dermoid cyst containing hair. The second was a case of pemphigus.

Dr. W. C. Lyle read a paper entitled "The Importance of Careful Chemical Analysis in Gastric Disease," dealing with the several tests used in rational diagnosis of disease of the stomach, and cited cases showing their value.

Dr. J. W. Duncan followed with a paper on "Germs in Health and Disease," emphasizing the importance of asepsis in operative work as well as the bacteriological relation of common diseases and public and private sanitation.

Dr. W. A. Elliott, of Savannah, then read a paper on "Mushrooms—a Food and a Poison." Considering the growing taste for mushrooms, it was well for the physicians to be acquainted with the symptoms of poisoning with this class of fungi. The edible fungi are classed as agarici, baleti, and puff-balls, though there are probably many others which may be included in the edible list.

He warned against all cupped mushrooms, especially if there are white gills. Reject those which are not perfectly fresh always.

Dr. Howard J. Williams, of Macon, read a paper entitled "A Supernumerary Cervical Rib: A Deception of Skiagraphy." The reader called attention to the fact that the skiagraph was not always an infallible guide, and cited two cases in which he had been misled into performing useless operations for apparent bone injury and a third case in which a supernumerary cervical rib showed as a bone tumor.

Dr. J. G. Hopkins, of Thomasville, read a paper on "Dislocation of the Sixth and Seventh Cervical Vertebrae, With General Paralysis," with report of such a case which terminated in recovery.

Dr. Hunter P. Cooper, of Atlanta, contributed a paper entitled "A Report of Surgical Cases," one concerned with the removal of a stone lodged in the urethra of a boy; another of amputation consecutive to railway injury in which reamputation was rendered necessary on account of the occurrence of osteo-sarcoma in the bone-stump.

Dr. Dunbar Roy, of Atlanta, presented a paper on "Peritonsilar Abscess," detailing the medical and surgical treatment of this affection. He highly praised local applications of strong silver nitrate solutions as an abortive measure.

Dr. Willis F. Westmoreland, of Atlanta, reported twenty-nine cases of tracheotomy, all of which were successful. He demonstrated the size of the trachea at various ages and exhibited specimens.

Dr. M. F. Carson, of Griffin, read a paper on "The Condition of Imperfect Septum Between the Mouth and Nasal Pharynx, Usually Termed Cleft Palate," in which he advised early closure of the fissure.

Dr. J. H. Shorter followed with a paper on "Suppurative Disease of the Middle Ear and Its Sequelæ," outlining the causes and course of otitis media purulenta.

Dr. J. M. Crawford, of Atlanta, read a paper on "Tonsillatomy, When and How to Make It." The operation should be resorted to when the tonsils extend much beyond the faucial pillars, and even in slight erypertrophy where there is a tendency to lacunar inflammation. The author prefers McKenzie's tonsillatome as being simple and easily managed. Hemorrhage is best controlled by pressure.

Dr. W. Z. Holliday, of Augusta, read a paper on "The Use of Ethyl Chloride as a Local Anæsthetic." He recommended it highly and said there was very little danger of the tubes exploding if carefully handled.

Dr. J. T. Ross, of Macon, presented a paper on "Ossific and Calcified Ovarian Fibroma," and exhibited specimen, which was covered with osseous deposit about one-sixteenth of an inch in thickness with numerous calcareous foci throughout the growth.

Dr. K. P. Moore, of Macon, read the report of a very interesting and "Unusual Monstrosity in the Form of Twins," and exhibited the specimen.

Dr. G. R. Carson, of Savannah, then read a paper on a "Rare Form of Bone Atrophy Following an Ununited Fracture, as Seen by the X-ray."

Dr. J. I. Griffith, of Danielsville, read a paper on "Puerperal Eclampsia, and Some of the Probable Causes of It."

Dr. G. H. Noble reported some successful cases of "Alcoholic Irrigation in Puerperal Sepris."

Dr. W. E. Fitch, of Savannah, read a paper on "Tight Lacing, Its Relation to Uterine Development and Diseases of the Female Organs of Generation," showing the deleterious effects of lacing and corset-wearing on the abdominal and pelvic viscera.

Dr. J. L. Hiers, of Savannah, then read a paper on "Malarial Asthenopia."

Dr. A. A. Davidson, of Augusta, read a paper on "Hysteria," with an analysis of the condition and the means of correcting it.

Dr. R. R. Kine, of Atlanta, then read a paper on "Medications for and Antiseptic Technique of Uterine Drainage After Labor and Abortion," in which he recommended drainage by means of tube and gauze wicking.

The following were elected officers for the ensuing year: President, Howard J. Williams, Macon; vice-president, J. G. Hopkins, Thomasville; second vice-president, Dr. I. H. Goss, Athens.

The association then adjourned to meet at Macon on the third Monday in April, 1899, which will mark the semi-centennial of the foundation of the association.

